# An Analytical Antenna Modeling of Electromagnetic Wave Propagation in Inhomogeneous Media Using FDTD: A Comprehensive Study

**DOI:** 10.3390/s23083896

**Published:** 2023-04-11

**Authors:** Dafnik Saril Kumar David, Yeongseok Jeong, Yin Chao Wu, Suyun Ham

**Affiliations:** Department of Civil Engineering, The University of Texas at Arlington, Arlington, TX 76019, USA

**Keywords:** GPR, ground-penetrating radar, EM wave, finite difference time domain

## Abstract

This paper discusses the challenges in characterizing electromagnetic (EM) waves propagating through inhomogeneous media, such as reinforced cement concrete and hot mix asphalt. Understanding the EM properties of materials, including their dielectric constant, conductivity, and magnetic permeability, is crucial to analyzing the behavior of these waves. The focus of this study is to develop a numerical model for EM antennas using the finite difference time domain (FDTD) method, and to gain a deeper understanding of various EM wave phenomena. Additionally, we verify the accuracy of our model by comparing its results with experimental data. We analyze several antenna models with different materials, including the absorber, high-density polyethylene and perfect electrical conductors, to obtain an analytical signal response that is verified against the experimental response. Furthermore, we model the inhomogeneous mixture of randomly distributed aggregates and voids within a medium. We verify the practicality and reliability of our inhomogeneous models using experimental radar responses on an inhomogeneous medium.

## 1. Introduction

Ground-penetrating radar (GPR) is a non-destructive investigative tool that uses electromagnetic (EM) waves and is commonly utilized in the fields of geophysics, structures, and transport systems [1,2,3]. However, the complexity of GPR data, which are based on EM waves, presents a challenge for the users attempting to interpret them. Interpreting EM wave data from GPR is often difficult and prone to errors due to the complex nature of the signals and the many factors that influence them. To successfully interpret these EM wave data, significant expertise is necessary, either through expensive experimentation or prior knowledge gained from costly mistakes in the field [4]. Therefore, analyzing and verifying these EM waves through simulations can play a role in securing the reliability of the GPR data analysis and assisting the expert knowledge of data analysts. Past simulations of EM waves, including models of actual antenna details, have primarily focused on the antennas used in academia or for research purposes [5,6,7,8,9,10,11,12,13,14,15,16,17]. The development of an FDTD-based bowtie antenna and a study on radar wave propagation have been carried out in previous works [5,6,7,8,9,10,11,12]. Their verifications were performed through comparisons of experiments and simulations [13,14,15,16,17]. Recent advancements in high-performance computing technology have made EM wave simulations increasingly useful within various fields. As a result, EM wave modeling is rapidly increasing in popularity, and the quality of GPR models is becoming more realistic [1,3].

EM wave simulations can be conducted using various numerical methods, including the finite difference time domain (FDTD) method, the finite element method (FEM), and the method of moments (MoM). In the FDTD method, the simulation domain is partitioned into a grid of small cells and the electric and magnetic fields at each cell are calculated at discrete time steps [18]. The FEM divides the simulation domain into small elements and uses interpolation functions to represent the fields within each element. The MoM expresses the fields in terms of a set of unknown currents on the surfaces of the objects in the simulation domain and uses numerical techniques to solve for these currents.

In a study conducted by Bai and Sinfield (2014), it was concluded that FEM is better suited to accurately describing complex geometries and the representing field. On the other hand, FDTD is better suited to spectroscopic studies when a large number of wavelengths are of interest and/or when a non-regular and/or non-Cartesian mesh refinement is implemented. Hence, the FDTD method is preferable for the simulation of EM waves under the same conditions, as well as for GPR simulations. Furthermore, recent computational advancements have significantly benefited volume-based numerical techniques such as the FDTD method and enabled larger and more complex problems to be investigated. This, combined with the need to obtain quantitative information from GPR, necessitates the creation and use of detailed 3D FDTD models of realistic GPR antennas [16].

In their recent study, [1] employed the FDTD method to numerically estimate the bulk permittivity of concrete. They then used this estimation to fine-tune the shape factor of the complex refractive index model (CRIM) model. Through laboratory measurements, they showed that the revised CRIM model outperformed the default shape factor and provided more accurate estimations of the bulk permittivity of the concrete. The authors created 3D concrete models for the simulation, with a 1.5 GHz center frequency GSSI-like antenna structure on the surface. However, the experimental framework that was used to validate the revised shape factor utilized a horn antenna with a central frequency of 1 GHz on top of a surface consisting of concrete blocks. This experimental validation could be improved by using the same antenna frequency that was used in the numerical analysis. Another recent study [3] used 3D modeling to simulate the behavior of concrete pavement with a random distribution, but for the computational efficiency, it only utilized the 2D slice of the 3D model. Additionally, the study used a transmitter and receiver as the sources for the EM wave simulation, which is not representative of commercial antennas. To improve the accuracy of the simulation verification, it would be beneficial to use a similar antenna model and consider the effects of the full 3D geometry.

To validate the results obtained through the use of random and irregular materials, it is crucial to match the various conditions during the experimental study of the inhomogeneous model. This paper presents works that were conducted to overcome the key technical barriers for a numerical analysis of the inhomogeneous model, with a model of the EM wave using a 1600 MHz antenna to conduct the verification experiment. The antenna model was constructed by conducting an experiment where the 1600 MHz antenna emitted EM waves between the antenna and a steel plate that was placed at a distance of 230–506 mm. The analysis model was then fine-tuned to match the experimental results obtained under the same conditions. This approach ensured that the experimental and numerical analyses were conducted under identical conditions, thus validating the accuracy of the results obtained through the numerical analysis. The findings indicate that the newly designed antenna model has potential for the analytical simulation of inhomogeneous random distributed particles. This model serves as a valuable reference for analysis, complementing the experimental results and improving the accuracy of GPR signal analyses in such media.

## 2. Theory

The FDTD method is a widely used numerical technique for simulating EM fields. It discretizes both the time and space domains and solves Maxwell’s equations at discrete points in the time and space to simulate the wave propagation. The governing equations for Maxwell’s time-varying EM fields are expressed in Equations (1)–(4).

(1) $$\nabla \cdot D=\rho_{v}$$

(2) ∇·B=0

(3) $$\nabla \times E=-\frac{\partial B}{\partial t}$$

(4) $$\nabla \times H=J+\frac{\partial D}{\partial t}$$

(5) D=εE

(6) B=μH

The electric flux density (D) is the propagation of the electric field (E) through a medium with permittivity (ε), while the magnetic flux density (B) is the propagation of the magnetic field (H) through a medium with permeability (μ). The charge density ($\rho_{v}$) and the current density (J) describe the distribution of the charge and current within the medium. To study a free-source medium, we can apply the curl operator to both sides of Equation (3).

(7) $$\nabla \times \nabla \times E=-\frac{\partial \nabla \times B}{\partial t}$$

By solving Equation (7), we can obtain the three-dimensional spatial effect resulting from the changes in the electric field over time, while taking into account the permittivity and permeability of the medium, as shown in Equation (8).

(8) $$\nabla^{2}E=\mu \varepsilon \frac{\partial^{2}E}{{\partial t}^{2}}$$

$$\nabla^{2}E=\frac{\partial^{2}E}{{\partial x}^{2}}+\frac{\partial^{2}E}{{\partial y}^{2}}+\frac{\partial^{2}E}{{\partial z}^{2}}$$

The FDTD method, which employs the second-order central difference algorithm in the Yee cell, is commonly used to simulate EM wave propagation. This numerical method involves discretizing both the space and time and replacing the derivatives in Ampere’s and Faraday’s laws with finite differences. By doing so, the electric and magnetic fields can be staggered in both space and time. The difference equations are then solved to obtain updated equations that express the future EM fields in terms of the past EM fields. The magnetic fields are evaluated one time-step into the future, effectively becoming past fields, followed by the evaluation of the electric fields one time-step into the future, also becoming past fields. These steps are repeated until the fields have been obtained over the desired duration. The FDTD method is a powerful numerical tool for simulating EM wave propagation and has applications in various fields, including optics, electromagnetics, and microwave engineering. Considering the Yee cell theory, the Maxwell equation with the applying curl can be rewritten, as shown in Equations (9)–(14).

(9) $$-\sigma_{m}H_{x}-\mu \frac{\partial H_{x}}{\partial t}=\frac{\partial E_{z}}{\partial y}-\frac{\partial E_{y}}{\partial z}$$

(10) $$-\sigma_{m}H_{y}-\mu \frac{\partial H_{y}}{\partial t}=\frac{\partial E_{x}}{\partial z}-\frac{\partial E_{z}}{\partial x}$$

(11) $$-\sigma_{m}H_{z}-\mu \frac{\partial H_{z}}{\partial t}=\frac{\partial E_{y}}{\partial x}-\frac{\partial E_{x}}{\partial y}$$

(12) $$\sigma E_{x}+\varepsilon \frac{\partial E_{x}}{\partial t}=\frac{\partial H_{z}}{\partial y}-\frac{\partial H_{y}}{\partial z}$$

(13) $$\sigma E_{y}+\varepsilon \frac{\partial E_{y}}{\partial t}=\frac{\partial H_{x}}{\partial z}-\frac{\partial H_{z}}{\partial x}$$

(14) $${\sigma E}_{z}+\varepsilon \frac{\partial E_{z}}{\partial t}=\frac{\partial H_{y}}{\partial x}-\frac{\partial H_{x}}{\partial y}$$

In the Yee cell numerical simulation, the parameters affecting the medium are taken into consideration. These parameters include the permittivity (ε) and permeability (μ) that are used in the Maxwell equations (Equations (1)–(4)), as well as the magnetic conductivity ($\sigma_{m}$) and electric conductivity (σ) of the medium. These properties affect the EM waves by influencing the wave energy’s attenuation and interface within the medium.

## 3. Method

This study consists of two main tasks. The first task aims to understand EM waves through different antenna modeling designs and establish a testing configuration for a comparative analysis of simulation models. The second task focuses on understanding the behavior of the EM waves through inhomogeneous mediums. Specifically, we aim to establish a randomly distributed particle (RDP) modeling approach and verify its accuracy through experimentation with aggregate specimens.

To achieve the first task, we will begin by conducting a comprehensive review of the existing literature on EM waves and antenna modeling. We will then design and create simulation models using different antenna designs to evaluate their effectiveness in generating and receiving these EM waves. These simulation models will be tested using a testing configuration that we will establish to enable a comparative analysis. To accomplish the second task, we will develop the RDP modeling approach based on its established principles and theory. We will create simulation models of the EM waves through the inhomogeneous medium using the RDP approach. To verify the accuracy of the RDP approach, we will perform experiments with aggregate specimens, comparing the results obtained from the simulation modeling with those obtained from the experimentation. Throughout both tasks, we will use analytical tools such as a numerical analysis and statistical analysis to analyze the data obtained from the experimentation and simulation modeling. This will enable us to draw conclusions and make recommendations based on our findings.

### 3.1. Testing Configuration for Comparative Analysis of Simulation Models

The methodology involved in this study utilizes the FDTD numerical method for EM wave propagation simulations. To accurately simulate the wave propagation, it is important to carefully consider the input sources used in the simulation. Therefore, various input source signals were studied in different setups and locations. These signals were then cross-verified with a reference signal obtained from a 1600 MHz antenna over an air medium. However, achieving an exact or even a similar match between the FDTD signal and the experimental signal is a challenging task. To address this issue, the study focuses on obtaining a signal match within the wave propagating medium. In order to validate the wave propagation within the air medium at specific depths, a reference metal plate was placed at the respective depths to obtain a perfect reflection of the EM wave. The placement and reflection of the reference metal plate can be observed.

**Figure 1:**
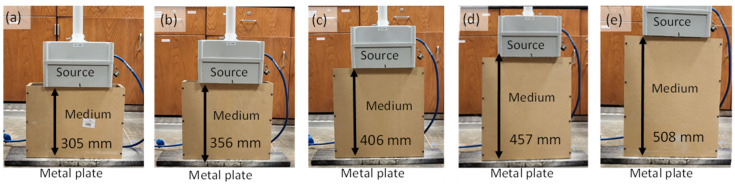
Experimental setup for scanning antenna at multiple depths: (**a**) 305 mm (12 inches), (**b**) 356 mm (14 inches), (**c**) 406 mm (16 inches), (**d**) 457 mm (18 inches), and (**e**) 508 mm (20 inches).

To validate the antenna model under varying conditions, the simulations were conducted at different depths of air medium, with a metal reference plate placed below each depth. The EM waves were propagated through the experimental setups at depths of 305, 356, 406, 457, and 508 mm (12, 14, 16, 18, and 20 inches), as depicted in Figure 1. The experimental setups were constructed using plywood with a width of 101 mm (4 inches) to minimize the impact on the wave propagation when it was scanned at the center. FDTD antenna models were also simulated for the experimental frequencies (1600 MHz) to examine the effects of the antenna and signal variability.

### 3.2. Modeling and Experimental Investigation of Randomly Distributed Particle

To achieve a random distribution of the particles without overlap, a Python code was developed to determine the 3D locations of the particles. Initially, equally spaced particles were modeled with the Python code, using Equations (15) and (16) to understand their patterns and locations.

(15) $$\begin{array}{c} {Nr}_{x}=L/r\\ {Nr}_{y}=B/r\\ {Nr}_{z}=H/r \end{array}$$

(16) $$\begin{array}{c} x=\left\{\begin{array}{c} L+\left(2\cdot \left({(Nr}_{x}/2)-i\right)+1\right)\cdot r,i\leq {(Nr}_{x}/2)\\ L-\left(2\cdot \left(i-{(Nr}_{x}/2)\right)+1\right)\cdot r,i>{(Nr}_{x}/2) \end{array}\right.\\ y=B-(2\cdot j-1)\cdot r\\ z=\left\{\begin{array}{c} H-(2\cdot \left(k-{(Nr}_{z}/2)\right)+1)\cdot r,k\leq {(Nr}_{z}/2)\\ H+\left(2\cdot \left({(Nr}_{z}/2)-k\right)+1\right)\cdot r,k>{(Nr}_{z}/2) \end{array}\right. \end{array}$$

**Figure 2:**
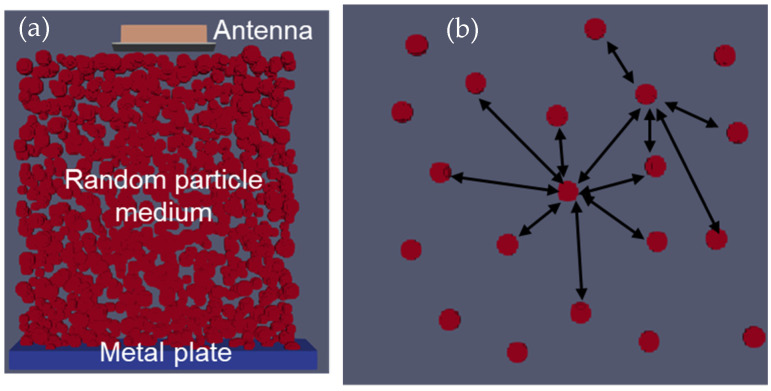
Simulation model of randomly distributed particles: (**a**) Developed from above algorithm, and (**b**) detailed aggregate model with tolerance for aggregate position.

To achieve a random distribution of the particles, the percentage of the total volume was taken into account, as shown in Equation (17), to determine the number of particles to be randomly placed in the model, with a tolerance distance between each particle, as specified in Equation (19). Once this tolerance distance was maintained, the respective particle sphere was added to the input file and the volume of the particle was reduced accordingly, as seen in Equation (20). An example of these randomly distributed particles is shown in Figure 2.

(17) $$V_{p}=Volume\;of\;Particle\;occupied\cdot l\cdot b\cdot h/100$$

(18) $$\begin{array}{c} V=\frac{4}{3}\pi r^{3},\\ x_{i}=random(x_{l},x_{u})\\ y_{i}=random(y_{l},y_{u})\\ z_{i}=random(z_{l},z_{u})\\ (x,y,z)\;\text{are}\;\text{the}\;\text{new}\;\text{coordinates} \end{array}$$

(19) $$d_{j}=\sqrt{{\left(x_{i}-x\right)}^{2}+{\left(y_{i}-y\right)}^{2}+{\left(z_{i}-z\right)}^{2}}-2r$$

$$\mathrm{Check}d_{j+1}>\mathrm{tolerance},\;\mathrm{then}\;\mathrm{location}\;\mathrm{and}\;\mathrm{radius}\;\mathrm{of}\;\mathrm{the}\;\mathrm{sphere}\;\mathrm{is}\;\mathrm{added}\;\mathrm{to}\;\mathrm{the}\;\mathrm{input}\;\mathrm{file}$$

(20) $$\mathrm{Reduce}\;\mathrm{the}\;\mathrm{volume},\;V_{p}=V_{p}-V$$

**Figure 3:**
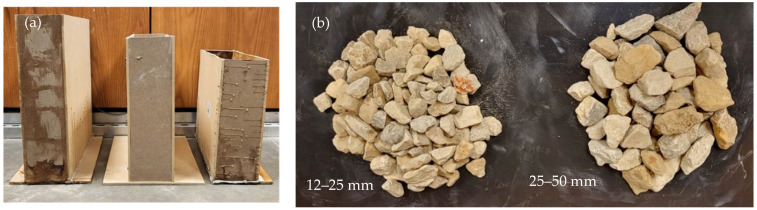
Experimental setup for (**a**) testing varying depth, with (**b**) two different types of aggregates of sizes 12–25 mm and 25–50 mm.

The random particle experiment was conducted at three different depths of 305, 356, and 405 mm (12, 14, and 16 inches), which were made of plywood and had two aggregate sieve size ranges of 12–25 mm and 25–50 mm (0.5–1 inch and 1–2 inches), as shown in Figure 3. Their comparison will be discussed in Section 4.3.

## 4. Result

### 4.1. Evaluating Parameters for the Refinement of Antenna Design

Various model parametric values were simulated to verify the accuracy of the input source and their results were compared with the experimental response, as shown in Table 1. In each study, the energy and travel times were evaluated, taking into account the boundaries of the medium and the metal reflection at the zero crossing before each peak reflection, respectively. The main focus of this study was to observe the time and signal attenuation within the medium, rather than the response of the initial direct wave. Consequently, only the reflected waves between the metal and medium, including the air and aggregate mediums, were considered. A metal plate served as a reference for the boundary of the medium. The zero crossing intersections were obtained using the Akaike information criterion (AIC) of the signal response data to determine the peak and low values within that time interval range. The energy of each signal was obtained from the area of the signal between the time interval of the medium and the metal reflection, while the travel time was evaluated from the time difference between the medium and the metal reflection.

**Figure 4:**
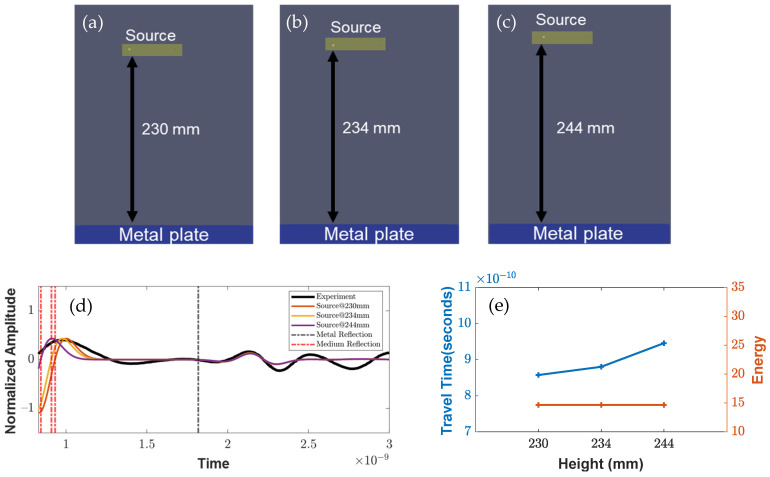
Simulation setup (from (**a**–**c**)) for various heights from 230 to 244 mm. (**d**) Wave responses for variable heights, and (**e**) parametric study of various heights with increasing travel time and similar energy.

**Figure 5:**
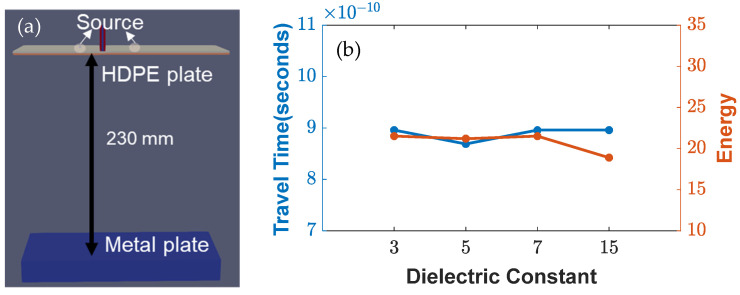
(**a**) Simulation setup for variable dielectric constants (3–15) considering a high-density polyethylene plate below the sensors, and (**b**) parametric study of various dielectric constants with similar travel times and energies.

**Figure 6:**
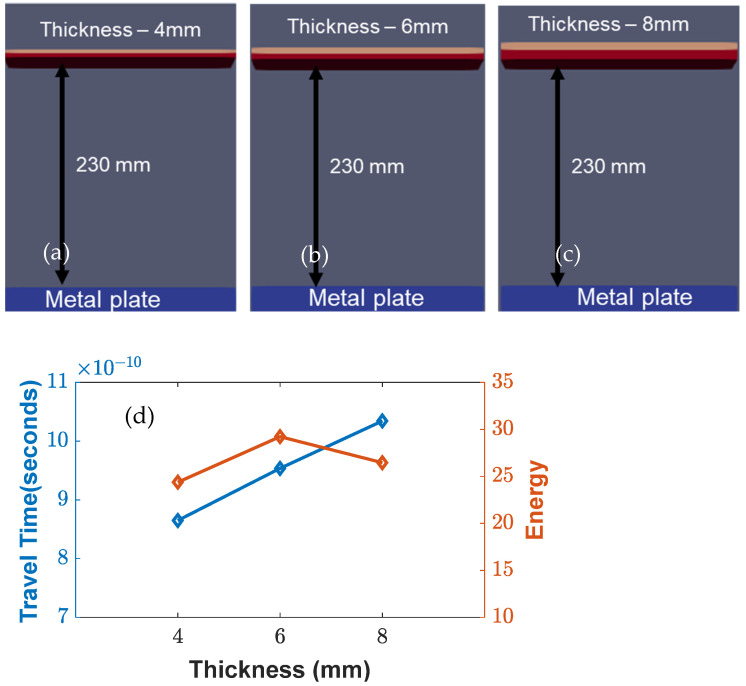
Simulation setup (from (**a**–**c**)) for variable thicknesses (4–8 mm) considered for printed circuit board and high-density polyethylene plate below the sensors, and (**d**) parametric study of various thicknesses with increasing travel times and variable energies.

The objective of this study was to investigate the response of EM waves under different parameters, in order to understand their effects and variability with each antenna design component. Figure 4, Figure 5 and Figure 6 present the results that were obtained from the conducted simulations. Figure 4d shows the comparison between the experimental response (represented in black) and the antenna setup, which only considered the transmitters and receivers of different heights through an air medium of a 230 mm (9-inch) depth. The study aimed to observe the effect of a variable height (0–14 mm) on the travel time and energy attenuation, with the center line in black indicating the boundary between the air medium and the metal plate. The results showed that an increase in the height led to an increase in the travel time, while the energy attenuation remained almost constant. These findings suggest that the height of the transmitter and receiver is a crucial factor that affects the travel time of EM waves and should be taken into consideration in the design of antenna systems.

Figure 5 was designed to examine the effect of various dielectric parameters on the travel time and energy attenuation of the EM waves. For this purpose, the transmitter and receiver were placed over high-density polyethylene (HDPE) plates of different dielectric constants, while a constant depth of 230 mm was maintained. The study found that an increase in the dielectric constant of the HDPE resulted in almost the same travel time, but the energy attenuation was reduced when higher dielectric constants were considered. Additionally, different antenna heights were considered to match the start of the medium.

In Figure 6, the effect of the plate thickness variation for both the high-density polyethylene (HDPE) and the printed circuit board (PCB) was analyzed. The results showed that an increase in the plate thickness led to an increase in the travel time, while the energy attenuation remained almost constant. This suggests that the thickness of the plate is an important factor in determining the travel time of the EM waves and should be considered in the design of antenna systems.

**Figure 7:**
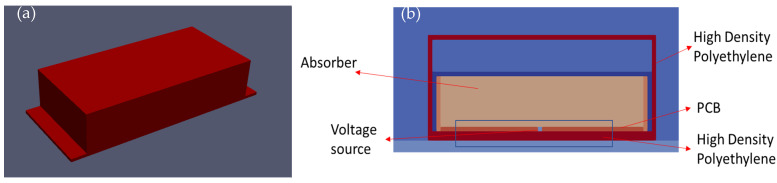
(**a**) Perspective view of the designed antenna model. (**b**) Cross-section of the designed antenna model.

The parameter studies conducted in this paper demonstrate that a simple source and transmitter cannot accurately represent a GPR signal response, and instead, the structure of the antenna must be taken into consideration. In this study, the internal components of the antenna module were considered from a cross-sectional perspective [16], with the antenna model confined between the plastic HDPE plates, as shown in Figure 7b. Absorbers and a perfect electric conductor were also utilized. These components were incorporated into the antenna model for the 1600 MHz frequency, along with the parameter studies. The resulting wave response closely resembled the experimental response that was observed between the medium and metal reflections. The thicknesses, dielectric constants, and conductivities of these internal components were determined through several simulations, and their final values for the antenna design setup are noted in Table 2.

### 4.2. Verification of the Antenna Model with Depth Variation

**Figure 8:**
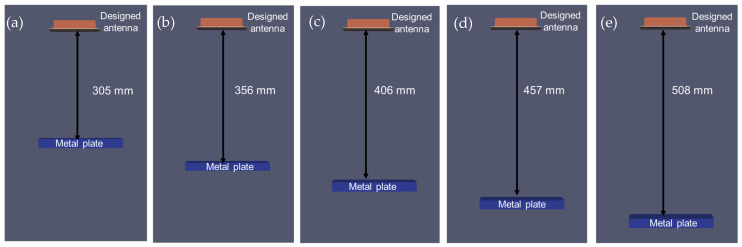
Simulation of the designed antenna at various depths: (**a**) 305 mm (12 inches), (**b**) 356 mm (14 inches), (**c**) 406 mm (16 inches), (**d**) 457 mm (18 inches), and (**e**) 508 mm (20 inches).

**Figure 9:**
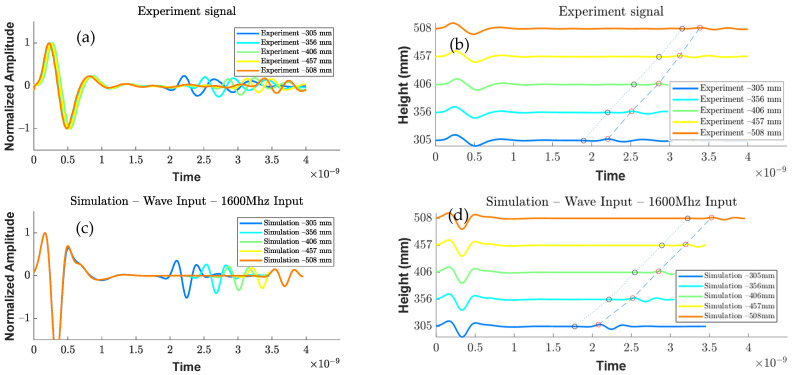
Data comparison: wave response analysis in air at different depths (305–506 mm) for (**a**) experiment and (**c**) simulation, and waterfall plots for peak amplitude and boundary of metal reflection from (**b**) experiment and (**d**) simulation.

**Figure 10:**
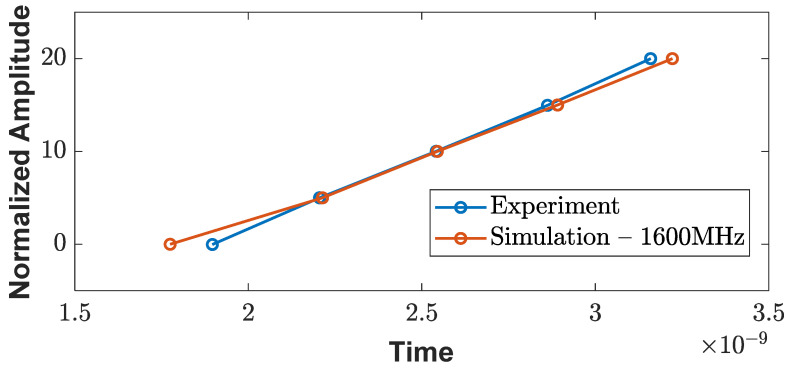
Comparison of slope lines similarity between simulation and experiment.

To validate the accuracy of the designed antenna, simulation models were created with varying depths of air medium (ranging from 305–506 mm or 12–20 inches), as shown in Figure 8. Similar experimental setups were performed to analyze the wave responses of the different depths of the air medium, as depicted in Figure 1. The wave responses of the experimental and simulation antenna designs are shown in Figure 9a,c. In both cases, a delay in the arrival time and a reduction in the amplitude were observed. In the waterfall plot of Figure 9b,d, the dotted line connects the arrival times of the metal plate and the metal reflection peaks, and the slope of these lines indicates a velocity change due to the varying depths. Upon comparing the experiment and simulation slope lines (Figure 10), their trends are similar and overlapping, indicating that the current antenna model design is highly accurate for the signal analysis.

### 4.3. Verification of the Inhomogeneous Medium with the Designed Antenna Model

**Figure 11:**
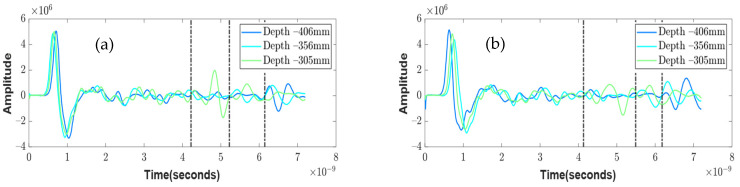
Experimental wave responses result of the different size of aggregates 12–25 mm (**a**), and 25–50 mm (**b**), at three different depths.

**Figure 12:**
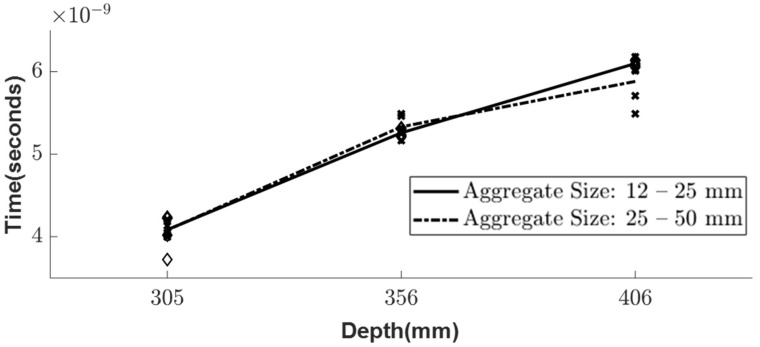
Scatter plot of the arrival time in experimental results for two different sizes of aggregates.

The experimental setup for testing the inhomogeneous aggregate–void medium is illustrated in Figure 3, and it was conducted for three different depths, namely 305, 356, and 406 mm. To achieve a greater accuracy in the simulation, two different aggregate sizes of 12–25 mm and 25–50 mm, which were obtained from the sieve analysis, were considered. As the depth increased, the arrival times of the experimental wave responses (Figure 11) showed delays in the wooden plate reflection. Since the medium was inhomogeneous, multiple wave response scans were recorded at five different locations for each setup. The arrival times of these different locations are shown as scatter plots in Figure 12, along with the average line connecting each depth.

**Figure 13:**
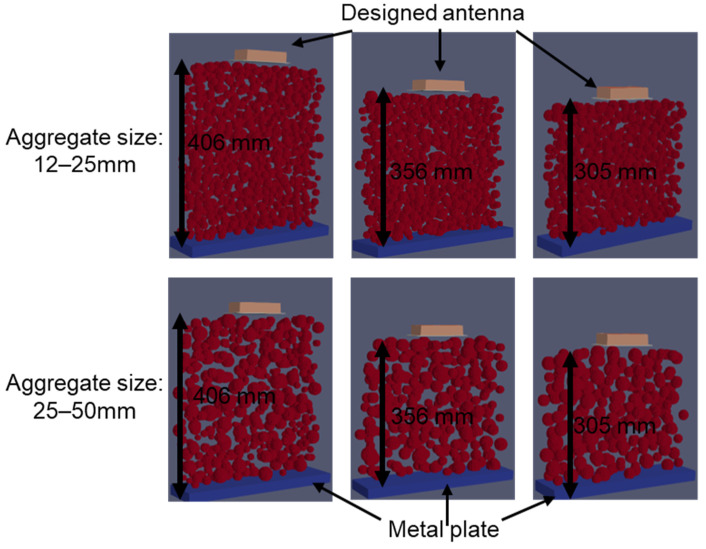
In homogenous simulation model generated from random aggregate particles for different depths at two different sizes of aggregates.

**Figure 14:**
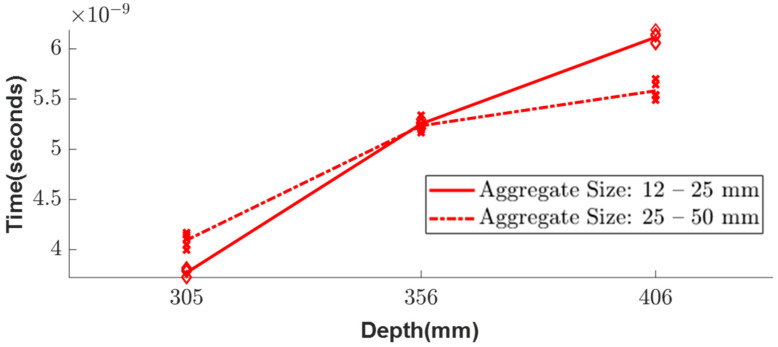
Scatter plot of the arrival time in simulation results for two different sizes of aggregates.

The simulation model was analyzed under the same conditions as the experimental setup shown in Figure 3. Using Python, the simulation of the inhomogeneous medium was modeled with the randomly generated aggregate particles and analyzed for the three different depths—305, 356, and 406 mm (Figure 13). The particles considered for this simulation were aggregates with known dielectric constants of 4.2, which was evaluated from the experiment. Similar to the experimental results, the simulation wave response shows a linear trend (Figure 14) when plotting the arrival times for the three different depths of both aggregate sizes.

**Figure 15:**
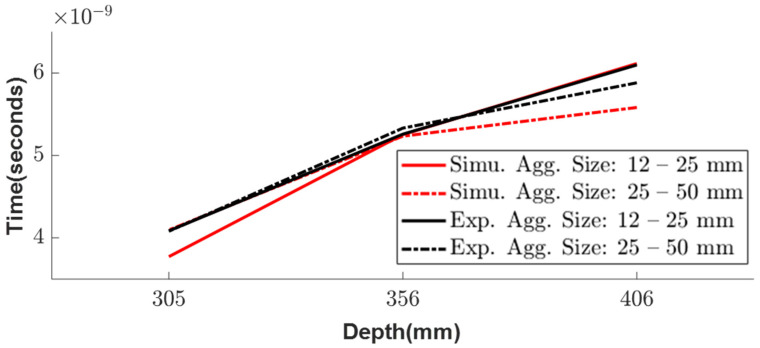
Comparison of time variations at different depths between simulation and experiment depth.

In comparing the results of the random particle simulation and the experimental study for the different sizes of aggregates, a similar trend is observed, but there is a delay in the arrival time at each depth due to the random distribution of the aggregate models and variable sizes (Figure 15). However, the simulation considers all the aggregates as spheres, which may not accurately represent the irregular shapes of the real aggregates. Therefore, further research is needed to study the inhomogeneous random aggregate patterns with irregular sizes to improve the accuracy of the simulation.

## 5. Conclusions

The use of the FDTD method has led to a better understanding of EM propagation, with the identification of the various factors that affect the medium, including the dielectric constant, dimensions, and sensor location. The development of a newly designed antenna model, similar to an experimental 1600 MHz antenna, has aided in investigating the EM wave propagation in response to different mediums. Based on our findings, the following conclusions can be drawn:

- The dielectric constant and dimensions of the material through which an EM wave propagates can affect the signal’s travel time and energy attenuation. However, for a more comprehensive analysis of the wet- and magnetic-related phenomena, other parameters such as conductivity and magnetic permeability must also be taken into account.
- A simple voltage source or receiver cannot be considered to be equivalent to an experimental antenna. To achieve a similar response to that of the experimental results, a well-designed antenna model should include absorbers, PCBs, and HDPE covers.
- The 1600 MHz antenna model that was designed showed reasonable analysis results when compared to the arrival time of the metal reflection in relation to the experimental propagation of the EM waves through the air medium.
- The simulation models that were developed using the randomly generated particle algorithm demonstrated a good comparison with the experimental results of the aggregate–void inhomogeneous medium when analyzed with the antenna model.
- Additionally, the randomly generated particle algorithm only considered a sphere shape for its analysis. Further study on the generation of irregular inhomogeneous mediums for a better analysis of the EM propagation needs to be considered.

## Figures and Tables

**Table 1:** Parameter variable considered for antenna model design.

Parameter	Value
Time window	4 × 10^−9^ s
Domain (x, y, z)	0.5 × 0.5 × 0.7 m
Spatial resolution (x, y, z)	0.002 × 0.002 × 0.002 m
Height (above the medium)	0–14 mm (with medium 230–244 mm)
High-density polyethylene with dielectric constant	3–15
Thickness of the HDPE/PCB	4–8 mm

**Table 2:** Parameter values of the antenna model obtained after several simulations.

Material	Dielectric Constant	Conductivity
High-density polyethylene	2.35	0
Absorber 1	3	0
Absorber 2	1.58	0.628
PCB	3	0

## Data Availability

Not applicable.
